# Laparoscopic assisted hydrocelectomy of the canal of Nuck: a case report

**DOI:** 10.1186/s40792-021-01137-3

**Published:** 2021-02-18

**Authors:** Liming Wang, Taku Maejima, Susumu Fukahori, Katayose Shun, Daitaro Yoshikawa, Toru Kono

**Affiliations:** Department of Surgery, Sapporo Higashi Tokushukai Hospital, 3-1, N-33, E-14, Higahi-ku, Sapporo, Hokkaido 0650033 Japan

**Keywords:** Hydrocele of canal of Nuck, Anterior approach, Laparoscopic assist

## Abstract

**Background:**

Accurate diagnosis and complete resection of hydrocele of canal of Nuck (HCN) is still a challenge for surgeons.

**Case presentation:**

A 28-year-old woman presented with a suspected inguinal hernia due to swelling in her right groin and was introduced for surgical treatment. Computed tomography scan revealed local cyst formation in the right groin and eliminated intestinal incarceration. In order to further confirm the diagnosis, we used laparoscopic exploration; after excluding a combined hernia, HCN was surgically removed using a conventional anterior peritoneal approach and a mesh patch repair was not needed. Postoperative pathology results showed no endometriosis or malignancy.

**Conclusions:**

Laparoscopic assisted anterior approach provides both an accurate intraoperative diagnosis and a quick complete resection of HCN; it is the preferred treatment for women of childbearing age with pure HCN.

## Background

Hydrocele of canal of Nuck (HCN) is a rare disease in adult women that is difficult to diagnose by preoperative imaging alone, and some patients may have a complicated inguinal hernia [1–3]. The traditional treatment method is to completely remove the hydrocele through an open anterior approach surgery [4, 5]. Although there are recent reports of cases of laparoscopic surgery, the anatomical location of the inguinal canal is deep, and it is also a challenge to strip the distal end of hydrocele through an inguinal canal [6–8]. Therefore, accurate diagnoses and removal of HNC quickly and completely remain a common problem faced by surgeons [9]. We report a case involving the diagnosis and rapid treatment of HCN using a combination of traditional and laparoscopic surgery.

## Case presentation

**Fig. 1:**
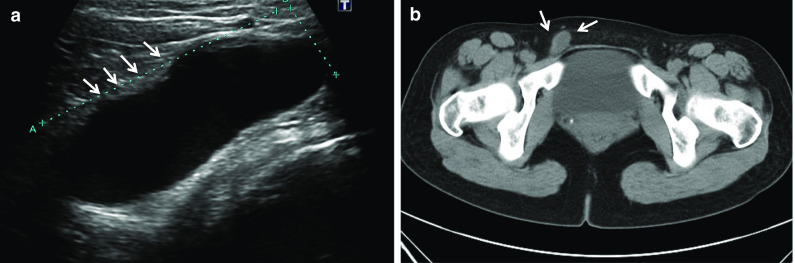
Preoperative imaging examination. **a** Ultrasound images revealed a hypoechoic fluid region in the right inguinal region. **b** Abdominal CT shows the right groin cyst, and no incarceration of the intestinal canal

A 28-year-old woman presented with a swelling in her right groin. She was suspected of having an inguinal hernia and was referred to surgery. There was no obvious enlargement of the mass when the abdomen was compressed in the standing position. Ultrasonography revealed a hypoechoic fluid region in the right inguinal region, with no blood flow (Fig. 1a). Computed tomography (CT) examination revealed cystic edema in the right groin and no incarceration of the intestinal canal (Fig. 1b). The patient was diagnosed as having HCN.

**Fig. 2:**
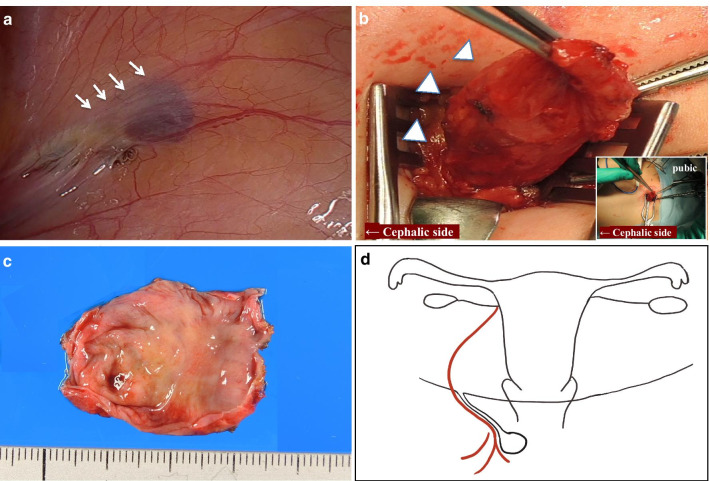
Intraoperative photos. **a** Laparoscope showed that there was a 1-cm liquid area in the ring in the right inguinal area, and the peritoneum is swollen by pressing the inguinal area in front (white arrows). **b** Complete excision of HCN by anterior approach (white arrowheads). **c** The groin cyst was filled with clear liquid and the wall was relatively smooth and flat. **d** Schematic illustration of right HCN

Considering that some patients may have a HCN combined with a hernia, we explored the abdominal cavity with a laparoscope. There was a 1-cm fluid area in the inner ring area of the right groin, which oppresses the front of the groin and swells the peritoneum (Fig. 2a). Although the inner ring is slightly weak, because there was no obvious hernia, we chose the anterior approach for tumor resection. We opened the external oblique muscle fascia to confirm that the HCN was free to the preperitoneal fat, ligated the root of the canal of Nuck at a high position (Fig. 2b, d), and performed a complete excision of the HCN. Finally, using laparoscopy, we reconfirmed there was no defect in the peritoneum. The operative time was 56 min. The patient recovered well and was discharged the next day.

**Fig. 3:**
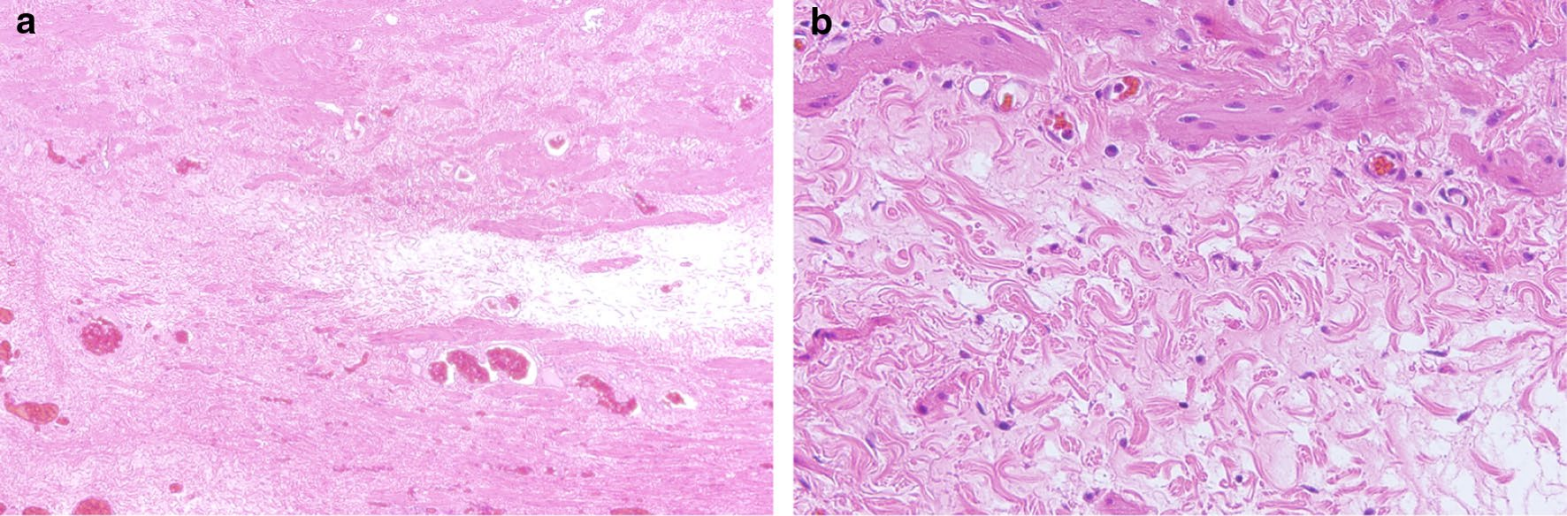
Pathological results. **a** Histopathology showing HCN accompanied by obvious congestion and mild inflammatory tissue (hematoxylin and eosin [HE], × 40). **b** There was no specific glandular tissue, endometrial tissue, or malignant cells ([HE], × 100)

Postoperative pathology showed that the size of the cyst was 4 × 4 cm. When the specimen was cut open, the capsule was filled with clear liquid and the wall was relatively smooth and flat (Fig. 2c). Hematoxylin and eosin-stained section showed HCN accompanied by obvious congestion and mild inflammatory tissue. There was no specific glandular tissue or endometrial tissue in the specimen, and no malignant cells were found (Fig. 3a, b).

## Discussion

In 1691, Dutch anatomist Anton Nuck first described HCN, which manifested as groin pain and compressible or incompressible local swelling of the labia [10, 11]. Unclosed HCN can cause asymptomatic effusion or hernia resulting in protrusion of abdominal organs, most commonly the intestine and ovaries [10]. This can lead to emergent situations such as strangulation obstruction of the intestine or torsion of the ovary. Additionally, part of the HCN may contain endometrial tissue, causing periodic swelling during menstruation [3, 12]. Due to these potential complications, timely diagnosis and prompt treatment of HCN is critical [8].

Imaging, especially ultrasound (US), is helpful for timely diagnosis; Doppler ultrasound can confirm intestinal obstruction and ischemic necrosis [5]. It has been reported that CT scan or magnetic resonance imaging can more effectively observe the anatomy around the cyst and determine whether the cyst communicated with the abdominal cavity [1]. Even so, in some cases, the final diagnosis depends on the intraoperative findings [4, 5]. Compared with the traditional anterior approach, the pneumoperitoneum in laparoscopic surgery will increase intra-abdominal pressure. Laparoscopy may be the best tool for diagnosing potential weak areas of the inner ring of the groin and can rule out the incarceration of internal organs in the abdominal cavity [13–15].

With the development of laparoscopy in recent years, there are related reports of laparoscopic removal of HCN, but HCN patients with indirect inguinal hernia can actively consider laparoscopic surgery [7, 16]. The inguinal hernia can be repaired at the same time. However, if it is only a simple HCN, laparoscopic removal of HCN will definitely lead to enlargement of the inner ring and a patch must be placed for repair [17].

For adult women of childbearing age, whether the patient has HCN combined with inguinal hernia, or the patient intends to be pregnant in the future [11, 14]. In addition, during laparoscopic HCN resection, it is difficult to successfully free the distal end of HCN because of the obstructed view of the deep inguinal canal and the inferior epigastric vessels [6, 7]. At this time, once laparoscopy finds that the patient has a pure HCN, the simplest anterior resection without hesitation may shorten the operation time [18].

Therefore, for the diagnosis and treatment of HNC, surgeons need to choose the best method according to the woman’s age, whether the patient has HCN combined with inguinal hernia, or the patient intends to be pregnant in the future. Laparoscopic combined with anterior approach undoubtedly provides the most accurate diagnostic method and the most rapid treatment for treatment of pure HCN. It may be considered as the preferred treatment method for young women of childbearing age without complicated hernia.

## Conclusion

Laparoscopic assisted anterior approach can not only provide accurate intraoperative diagnosis, but also a quick complete resection of HCN, which is the preferred treatment for women of childbearing age with pure HCN.

## Data Availability

The datasets supporting the conclusions of this article are included within the article and its additional files.

## Abbreviations

US: Ultrasonography.
CT: Computed tomography.
HCN: Hydrocele of canal of Nuck

## References

[1] Prodromidou A, Paspala A, Schizas D, Spartalis E, Nastos C, Machairas N (2020). Cyst of the Canal of Nuck in adult females: a case report and systematic review. Biomed Rep.

[2] Counseller VS, Black BM (1941). Hydrocele of the Canal of Nuck: report of seventeen cases. Ann Surg.

[3] Basnayake O, Jayarajah U, Seneviratne SA (2020). Endometriosis of the inguinal canal mimicking a hydrocele of the canal of Nuck. Case Rep Surg.

[4] Kim KS, Choi JH, Kim HM, Kim KP, Kwon YJ, Hwang JH (2016). Hydrocele of the Canal of Nuck in a female adult. Arch Plast Surg.

[5] Khanna PC, Ponsky T, Zagol B, Lukish JR, Markle BM (2007). Sonographic appearance of canal of Nuck hydrocele. Pediatr Radiol.

[6] Kojima S, Sakamoto T (2020). Laparoscopic total extraperitoneal treatment for a hydrocele of the canal of Nuck located entirely within the inguinal canal: a case report. Asian J Endoscopic Surg.

[7] Chihara N, Taniai N, Suzuki H, Nakata R, Shioda M, Yoshida H (2020). Use of a novel open posterior wall technique for laparoscopic excision of hydrocele of the canal of Nuck in an adult female: case report. J Nippon Med Sch.

[8] Chan D, Kwon JK, Lagomarsino EM, Veltkamp JG, Yang MS, Pfeifer CM (2019). Canal of Nuck hernias. Acta Radiol Open.

[9] Topal U, Saritas AG, Ulku A, Akcam AT, Doran F (2018). Cyst of the canal of Nuck mimicking inguinal hernia. Int J Surg Case Rep.

[10] Nasser H, King M, Rosenberg HK, Rosen A, Wilck E, Simpson WL (2018). Anatomy and pathology of the canal of Nuck. Clin Imaging.

[11] Yen CF, Wang CJ, Lin SL, Chang PC, Lee CL, Soong YK (2001). Laparoscopic closure of patent canal of Nuck for female indirect inguinal hernia. J Am Assoc Gynecol Laparosc.

[12] Uno Y, Nakajima S, Yano F, Eto K, Omura N, Yanaga K (2014). Mesothelial cyst with endometriosis mimicking a Nuck cyst. J Surg Case Rep..

[13] Cheng EM, Sarkar A, Perera DS (2020). Hydrocoele in the canal of Nuck in an adult female: a rare cause for inguinal swelling. ANZ J Surg..

[14] Fikatas P, Megas IF, Mantouvalou K, Alkatout I, Chopra SS, Biebl M (2020). Hydroceles of the Canal of Nuck in adults-diagnostic, treatment and results of a rare condition in females. J Clin Med..

[15] Matsumoto T, Hara T, Hirashita T, Kubo N, Hiroshige S, Orita H (2014). Laparoscopic diagnosis and treatment of a hydrocele of the canal of Nuck extending in the retroperitoneal space: A case report. Int J Surg Case Rep.

[16] Qureshi NJ, Lakshman K (2014). Laparoscopic excision of cyst of canal of Nuck. J Minim Access Surg.

[17] Shahid F, El Ansari W, Ben-Gashir M, Abdelaal A (2020). Laparoscopic hydrocelectomy of the canal of Nuck in adult female: Case report and literature review. Int J Surg Case Rep.

[18] Kohata A, Hirata Y, Ishikawa S, Kai A, Namba Y, Okimoto S (2020). Large hydrocele of the canal of Nuck diagnosed and treated using conventional and laparoscopic methods. J Surg Case Rep..

